# Hyperbaric oxygen therapy as an adjunct to corticosteroid treatment in sudden sensorineural hearing loss: a retrospective study

**DOI:** 10.3389/fneur.2023.1225135

**Published:** 2023-07-05

**Authors:** Piotr H. Skarzynski, Aleksandra Kolodziejak, Elżbieta Gos, Magdalena B. Skarzynska, Natalia Czajka, Henryk Skarzynski

**Affiliations:** ^1^Department of Teleaudiology and Screening, World Hearing Center, Institute of Physiology and Pathology of Hearing, Kajetany/Warsaw, Poland; ^2^Heart Failure and Cardiac Rehabilitation Department, Faculty of Medicine, Medical University of Warsaw, Warsaw, Poland; ^3^Institute of Sensory Organs, Kajetany/Warsaw, Poland; ^4^Center of Hearing and Speech Medincus, Kajetany/Warsaw, Poland; ^5^Pharmacy Department, Department of Pharmacotherapy and Pharmaceutical Care, Medical University of Warsaw, Warsaw, Poland; ^6^Otorhinolaryngosurgery Clinic, World Hearing Center, Institute of Physiology and Pathology of Hearing, Kajetany/Warsaw, Poland

**Keywords:** sudden sensorineural hearing loss, hyperbaric oxygen therapy, glucococorticoids, HBOT, hearing loss

## Abstract

**Background:**

A retrospective clinical study was conducted to test the impact of including hyperbaric oxygen therapy in the treatment of patients with sudden sensorineural hearing loss (SSNHL).

**Materials and methods:**

A total of 63 adult patients with sudden sensorineural hearing loss diagnosed between 2015 and 2023 were divided into two groups: 36 patients treated with intratympanic glucocorticoid and orally administered glucocorticoid who also underwent hyperbaric oxygen therapy and 27 patients treated with intratympanic glucocorticoid and prolonged orally administered glucocorticoid (without hyperbaric oxygen therapy). An audiological evaluation was performed using pure-tone audiometry.

**Results:**

Average hearing gain as measured by pure tone average was 12.5 dB HL (+/- 19.9 dB HL) in the patients treated with steroids combined with HBOT, and was 14.1 dB HL (+/- 17.9 dB) in the patients treated with steroids alone. Successful treatment (complete recovery or marked improvement) was observed in 27.8% of the patients in the first group and in 25.5% in the second group. There was no statistically significant difference between the groups.

**Conclusion:**

Both groups of patients—those treated with glucocorticoids and those treated with glucocorticoids and HBOT—had similar hearing outcomes. A prospective, controlled, and randomized study would provide more reliable knowledge about the efficacy of HBOT in treating SSNHL.

## 1. Introduction

Sudden idiopathic loss of hearing is called sudden sensorineural hearing loss (SSNHL). It is defined as a rapid deterioration in hearing that happens in up to 72 h in one or, less frequently, both ears. The audiometric criterion is a hearing deterioration of at least 30 dB at three contiguous frequencies (1, 2). Corticosteroid therapy is the primary method of intervention in the treatment of SSNHL. However, the treatment of patients varies because of its unknown etiology (3, 4).

Hyperbaric oxygen therapy (HBOT) involves breathing 100% oxygen at elevated ambient pressure (5). HBOT increases the partial pressure of oxygen in the tissues, in this case in the cochlea, which is an organ that is very sensitive to hypoxia. Oxygen reaches the spiral organ in two ways. First, by diffusion from the vascular stria through the endolymph of the cochlear duct, and second, by diffusion from the space of the middle ear through the membrane of the round window. The growth occurring during hyperbaric oxygen therapy in the fluids of the inner ear is accompanied by a rapid return of electrophysiological activity cochlea, which is crucial for its physiological activity (6). HBOT has complex effects on the cellular immune mechanism, oxygen transport, and hemodynamics, reducing tissue hypoxia and edema and modifying the patient’s response to infection and hypoxia (7). As a medical procedure, HBOT is carried out in special centers in an ambulatory mode. It usually involves 10–15 daily sessions that last 60 min each within a chamber with pressure ranging from 2.2 to 2.5 ATA. Early use of HBOT, within the first 2 weeks of SSNHL onset, may yield the best results for patients, and young patients generally benefit more than older patients [those over 50 years old; (8)]. Apart from impairments of the pneumothorax, there are no absolute contraindications to the use of a hyperbaric chamber, but a careful assessment of the potential therapeutic benefits relative to the risk in patients with claustrophobia, who are pregnant, or have cardiovascular diseases or implanted devices (e.g., pacemakers) should be carried out. According to current indications, before introducing HBOT, a cardiac ECG (electrocardiography) and chest X-ray should be performed on the patient (9). There is a small risk of side effects, but these should be taken into account. Side effects include barotrauma of the middle ear or paranasal sinuses, barotrauma of the lungs, or transient visual impairment (10).

The present study aimed to find out whether HBOT, added to steroid treatment, provides additional benefits. In other words: will the hearing improvement in patients treated with corticosteroids and HBOT be greater than in patients treated with corticosteroids alone?

## 2. Materials and methods

### 2.1. Ethical approval and patients studied

The protocol of this retrospective study was approved by the Bioethics Committee of the Institute of Physiology and Pathology of Hearing (IFPS:KB/Statement no. 17/2021). The inclusion criteria were: age above 18 years, hearing loss of sudden onset, and hearing loss of at least 30 dB at three contiguous frequencies. Exclusion criteria were: age below 18 years, no follow-up audiogram, and contraindications to treatment in a hyperbaric chamber. The study is a retrospective analysis of medical records gathered between January 2015 and January 2023.

### 2.2. Audiological assessment

An audiological evaluation was performed using pure tone audiometry. The test was conducted across 11 frequencies (0.125–8 kHz) using octaves. Pure tone audiometry was performed on the day the patient came to the clinic and at a follow-up visit. Measurements were carried out in the soundproof cabin using a diagnostic audiometer with calibrated earphones. Pure tone average (PTA4) was calculated using four frequencies: 0.5, 1, 2, and 4 kHz.

The criterion for hearing improvement was adopted according to Labatut et al. (11):

- complete recovery: PTA < 25 dB HL;
- marked improvement: PTA improvement of >30 dB HL;
- slight improvement: PTA improvement between 10 and 30 dB HL; and
- non-recovery: PTA improvement <10 dB HL.

Complete recovery or marked improvement = successful treatment.

### 2.3. Corticosteroids dosage

Dexamethasone (brand name Dexaven®, solution for injection, concentration 4 mg/mL) and prednisone (brand name Encorton®, tablets, 1, 5, 10, and 20 mg per tablet) were administered to the patients. Dexamethasone, at a maximum dose of 2 mL (8 mg), was administered to patients once. Prednisone, at a dose of 1 mg/kg body weight/24 h, was administered orally in the morning. In this study, dexamethasone was administered intravenously (between 4 and 16 mg/24 h) and as an intratympanic injection via drain at the maximum volume 0.3 mL (1.2 mg of dexamethasone) (4). The scheme of administration was as follows: between day 1 and 14: 60 mg of prednisone/24 h, after which the dose was reduced by 5 mg each day (so as to prevent adrenal insufficiency). Thus, on day 15, the daily dose was 55 mg; day 16, 50 mg; day 17, 45 mg; and so on until at day 25 when the dose was just 5 mg. In both groups, the steroid treatments were the same.

### 2.4. Session in a hyperbaric chamber

Hyperbaric oxygen treatment is the only known method of increasing the partial pressure of oxygen (pO_2_) in the fluids of the inner ear and as a result, it is used in the treatment of SSNHL. The decrease in pO_2_ in the inner ear is one of the end effects of damaging factors on the cochlea. A single session in the hyperbaric chamber lasts approximately 60–80 min and consists of three stages (12):

1. Slow compression phase (so-called “immersion”), when pressure is increased in the chamber. This phase lasts from 6 to 12 min until the target pressure of 2.5 ATA is reached.
2. The main stage lasts approximately 60 min. The chamber has the right pressure that allows oxygen to penetrate the blood, which supports the immune mechanisms and accelerates the regeneration of damaged tissues and organs. There are 10-min breaks between oxygen delivery periods.
3. Slow decompression phase, which consists in equalizing the pressure in the hyperbaric chamber. It lasts from 5 to 15 min.

### 2.5. Participants

There were two groups of patients: (1) 36 patients who were treated with intratympanic glucocorticoid, orally administered glucocorticoid, and who underwent hyperbaric oxygen therapy; and (2) 27 patients who were treated with intratympanic glucocorticoid and prolonged orally administered glucocorticoid (without hyperbaric oxygen therapy). The first group consisted of 21 women and 15 men aged between 24 and 70 years, with a mean age of 49.6 years (*SD* = 14.3). The second group consisted of 15 women and 12 men aged between 18 and 84 years, with a mean age of 49.7 years (*SD* = 18.0).

### 2.6. Statistical analysis

The demographic and clinical characteristics of the patients were examined using descriptive statistics and percentages. Change in hearing thresholds obtained before and after treatment was assessed through a Wilcoxon test for paired samples. The relationship between the variables was assessed using a *χ*2 test and Spearman’s rho coefficient. Statistical significance was set as a value of *p* of less than 0.05. The analysis was performed using IBM SPSS Statistics (version 24).

## 3. Results

Table 1 shows the clinical characteristics of the patients.

**Table 1:** Clinical characteristics of the patients.

		Group 1 (*n* = 36)	Group 2 (*n* = 27)
Ear with SSHL	Right	14 (38.9%)	8 (29.6%)
	Left	22 (61.1%)	19 (70.4%)
Feeling of fullness in the ear	Yes	11 (30.6%)	9 (33.3%)
	No	25 (69.4%)	18 (66.7%)
Tinnitus	Yes	22 (61.1%)	21 (77.8%)
	No	14 (38.9%)	6 (22.8%)
Vertigo/dizziness	Yes	10 (27.8%)	8 (29.6%)
	No	26 (72.2%)	19 (70.4%)
Treatment delay (days)	Range	1–13	0–16
	M (SD)	4.4 (3.5)	5.5 (3.9)
Follow-up visit (days)	Range	4–235	6–197
	M (SD)	65.9 (64.8)	38.1 (46.1)
Degree of hearing loss	Mild	5 (13.9)	8 (29.6)
	Moderate	18 (50.0)	10 (37.0)
	Severe	9 (25.0)	8 (29.6)
	Profound	4 (11.1)	1 (3.8)

Treatment delay is the time interval (in days) from the onset of symptoms to the initiation of treatment.

### 3.1. Hearing improvement after treatment

Pure tone average was calculated in both groups of patients. In the first group, before treatment, it was *M* = 63.3 dB HL (*SD* = 22.0), and after treatment, it was *M* = 50.8 dB HL (*SD* = 29.4); the change was statistically significant: *Z* = 3.24; *p* = 0.001 and the mean improvement was 12.5 dB HL (+/- 19.9 dB). In the second group, PTA4 before treatment was *M* = 56.8 dB HL (*SD* = 20.2), and after treatment, it was *M* = 42.7 dB HL (*SD* = 24.3); the change was statistically significant: *Z* = 3.64; *p* < 0.001 and the mean improvement was 14.1 dB HL (+/- 17.9 dB).

**Figure 1:**
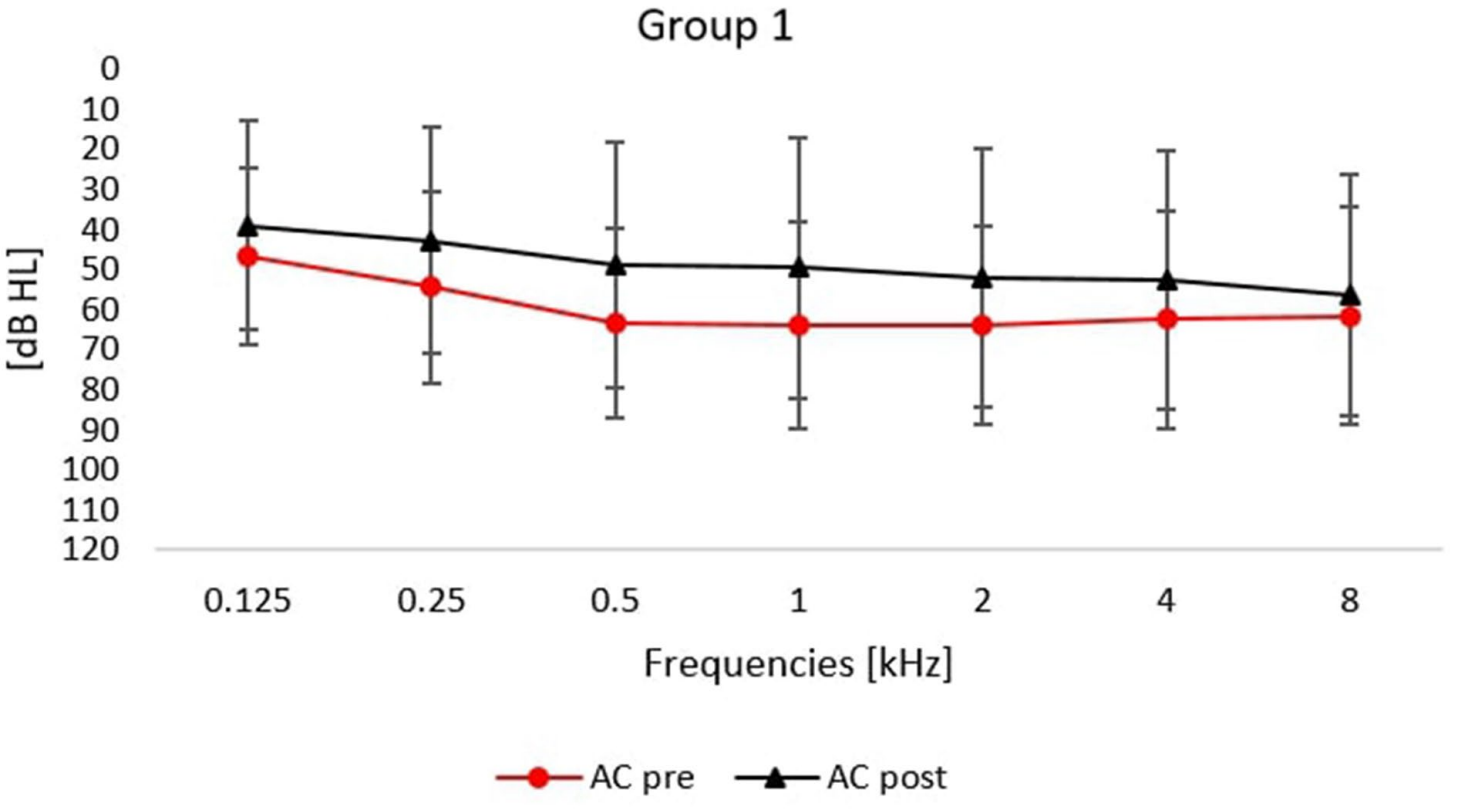
Averaged air conduction thresholds (pre- and post-treatment) for patients treated with intratympanic glucocorticoid, orally administered glucocorticoid and who underwent hyperbaric oxygen therapy. Error bars represent standard deviations.

**Figure 2:**
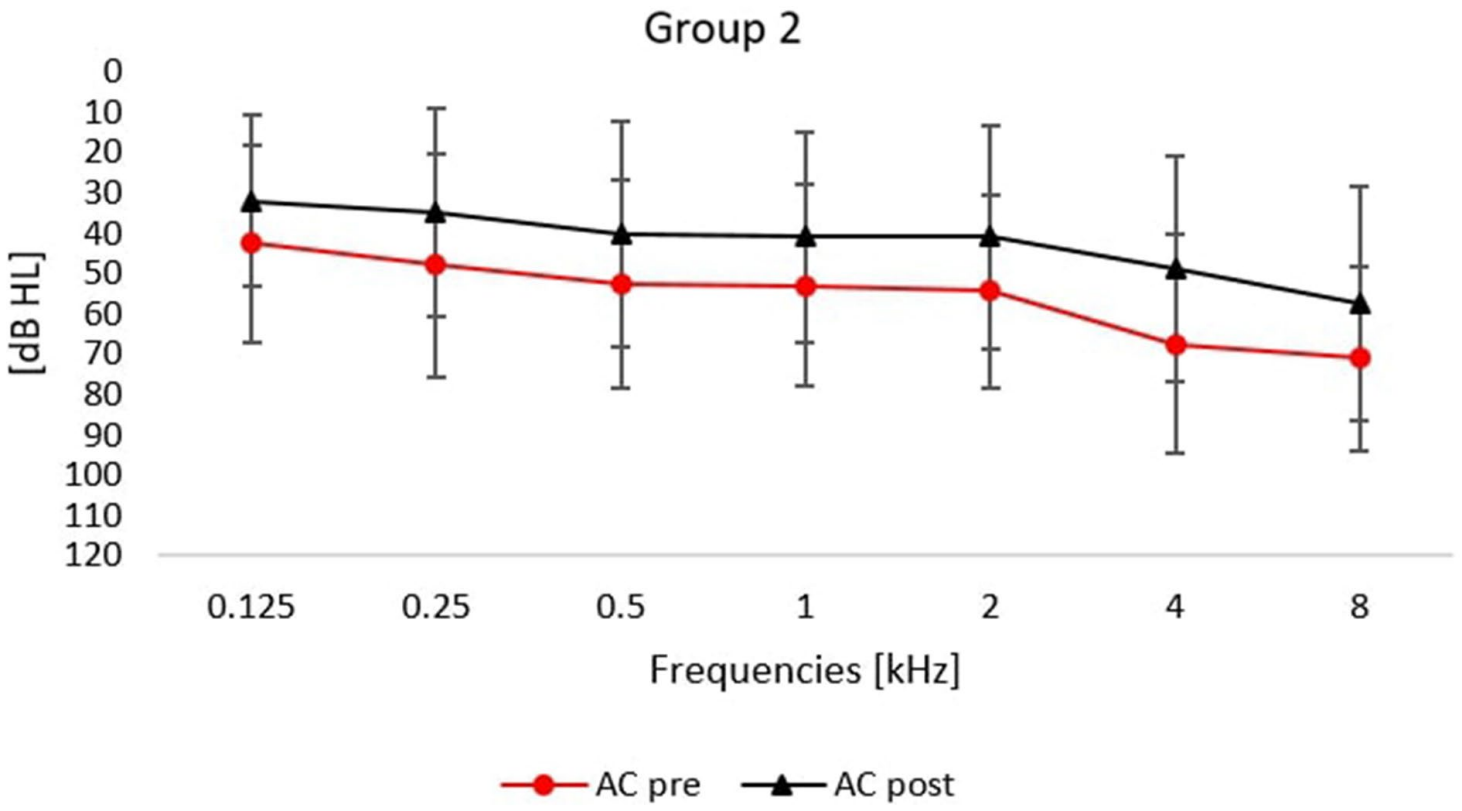
Averaged air conduction thresholds (pre- and post-treatment) for patients treated with intratympanic glucocorticoid and orally administered glucocorticoid (without hyperbaric oxygen therapy). Error bars represent standard deviations.

Detailed changes in frequencies from 0.125 to 8 kHz are shown in Figures 1, 2.

Table 2 shows hearing improvement after treatment in both groups of patients.

**Table 2:** Improvement after treatment.

	Group 1 (*n* = 36)	Group 2 (*n* = 27)
Complete recovery	8 (22.2%)	6 (22.2%)
Marked improvement	2 (5.6%)	1 (3.7%)
Slight improvement	8 (22.2%)	8 (29.6%)
Non-recovery	18 (50.0%)	12 (44.5%)

As can be seen in Table 2, hearing improvement in both groups was similar. Successful treatment (complete recovery or marked improvement) was observed in 27.8% of the patients in the first group and in 25.5% in the second group. The rates of slight improvement or non-recovery were also similar in both groups. The difference between the groups was not statistically significant: *χ*^2^ = 0.54; *p* = 0.909.

### 3.2. Relationship between treatment delay and hearing improvement

The correlation between treatment delay and change in PTA4 was *rho* = −0.14; *p* = 0.287. It was a negative correlation, so the longer the delay, the smaller the improvement, but the correlation was weak and statistically non-significant.

## 4. Discussion

The present study aimed to investigate whether HBOT, used in addition to glucocorticoid treatment for SSNHL, provided additional benefits in terms of hearing improvement. It is known that HBOT increases the oxygen concentration in the inner ear fluids, nourishes delicate structures of the cochlea, and supports its functioning, and in this way, it might help restore hearing.

In 2016, the European Committee on Hyperbaric Medicine updated indications for HBOT in various conditions, including in cases of sudden deafness. The recommendation for using HBOT in sudden deafness rates is level 1 (i.e., a strong recommendation) and comes from level B of evidence (i.e., a moderate level) (13). Many studies have shown that HBOT is beneficial in the treatment of SSNHL as an adjunctive therapy (14–16). In combination with glucocorticosteroids, it has been found to promote hearing gain (17, 18).

We did not find a beneficial effect of HBOT in our study. The results obtained by patients treated with glucocorticoids and HBOT were similar to those treated with glucocorticoids alone. The mean change in hearing thresholds was 12.5 dB HL in the first group and 14.1 dB HL in the second group. The rates of successful treatment were also similar, reaching 27.8% of the patients in the first group and 25.5% in the second group.

The results obtained by other researchers vary widely. Krajcovicova et al. (18) used HBOT as a supplement to the first line of medical treatment (steroids intravenously and orally) and compared the steroids alone condition and steroids with HBOT. They found that both protocols yielded hearing gain in patients with SSNHL, but HBOT significantly increased the effect of pharmacotherapy. The proportion of patients with hearing gain was 28.6% in those treated with steroids alone and 61.7% in those with combined treatment. The authors concluded that HBOT was a promising modality of SSNHL treatment.

Similar results were obtained in a randomized controlled trial by Cho et al. (17). Patients were divided into two groups: 30 treated with steroids alone (orally and intratympanically) and 30 with steroids and HBOT. The group treated with steroids and HBOT achieved significantly better hearing levels at 0.5 and 1 kHz (although the pure tone averages were similar in both groups), and the rate of hearing recovery was significantly higher (60 vs. 33%). Importantly, speech recognition improved more in patients with combined treatment than in patients treated with steroids alone.

An interesting study of the use of HBOT in the treatment of SSNHL was conducted by Liu et al. (19), and it sheds light on the molecular mechanism behind HBOT therapy. The study was performed on 120 subjects with idiopathic SSNHL, divided into two groups. The first group consisted of 60 patients treated with oral prednisone and ginaton only; the second consisted of 60 patients who received both medicine (oral prednisone and ginaton) and HBOT. Complete recovery or improvement was found in 66.7% of the patients in the medicine-only group and in 86.7% of the HBOT group. Additionally, inflammatory cytokines toll-like receptors (TLR4), nuclear factor (NF-κB) expression, and tumor necrosis factor (TNF-α) levels in the peripheral blood were measured. Reduced inflammation was observed after treatment in both groups, but hearing recovery was greater for those who received medicine and HBOT. Higher expression of toll-like receptor (TLR) genes in peripheral blood leukocytes of SNNHL patients has been confirmed by Yang et al. (20).

On the other hand, some researchers fail to confirm the beneficial effects of HBOT in SSNHL and point out that evidence is still scarce (21). Yücel and Özbuğday (22) compared two treatment regimens: steroids only (intravenously and intratympanically) and steroids with HBOT. In the steroid group, the rate of hearing recovery was 73.1%; in the steroid and HBO group it was similar, 69.1%. The authors concluded that there was no favorable effect of HBOT when added to steroid treatment. Skarżyńska et al. (4) compared five different pharmacological and non-pharmacological treatment regimes for SSNHL in which two had HBOT supplementation to glucocorticoid administration. The best outcomes were achieved by patients given intratympanic glucocorticoid combined with prolonged orally administered glucocorticoid. The inclusion of HBOT did not translate into better hearing results. Based on the results from this study and a previous one (4), it seems that the anti-inflammatory power and the route of administration of glucocorticoids are important. The cumulative dose of glucocorticoids (orally administered prednisone and locally administered dexamethasone) seems to play an important role in restoring hearing after an SSNHL incident. However, the anti-inflammatory power of dexamethasone in both groups of patients in this study appeared to be the primary factor in restoring hearing after an SSNHL incident.

Systematic reviews and meta-analyses can help resolve discrepancies in the effectiveness of HBOT in treating SSNHL (23, 24). A large clinical study by Rhee et al. (24) showed a beneficial effect of HBOT when used as part of a combination treatment, with the odds ratios for hearing recovery being significantly higher for the combined treatment. In a meta-analysis by Joshua et al. (23), there was a significant advantage in hearing gain and odds ratio for hearing recovery when HBOT was added to the treatment.

Given the above, one can ask why the results of our study did not favor HBOT treatment. We suggest that treatment delay may be a relevant factor here. Our patients reported to the clinic up to 2 weeks after the onset of SSNHL. We found there was a small negative correlation between treatment delay and the improvement in hearing thresholds, and this suggests it is more beneficial to begin treatment of SSNHL immediately.

There are several limitations to our study. Its design was retrospective and non-randomized, so the characteristics of the patients might affect the outcomes, meaning that any conclusions must be limited. In addition, follow-up visits took place at different times after treatment, so the results obtained after a prolonged time might be affected by unknown factors.

## 5. Conclusion

We did not find a difference in hearing outcomes between patients treated with glucocorticoids and those treated with glucocorticoids in combination with HBOT. A prospective, controlled, and randomized study would provide more reliable knowledge of the role of HBOT in treating SSNHL.

## Data availability statement

The raw data supporting the conclusions of this article will be made available by the authors, without undue reservation.

## Ethics statement

The studies involving human participants were reviewed and approved by Bioethics Committee of the Institute of Physiology and Pathology of Hearing (IFPS:KB/Statement no. 17/2021). Written informed consent for participation was not required for this study in accordance with the national legislation and the institutional requirements.

## Author contributions

PS and HS: conceptualization and supervision. AK: data curation. EG: formal analysis. PS, AK, MS, and EG: investigation. EG and AK: methodology and visualization. PS: project administrator and resources. PS, AK, EG, MS, and NC: writing original draft. PS, EG, AK, MS, NC, and HS: writing—review and editing. All authors contributed to the article and approved the submitted version.
